# IUC26690-88 Cannabinoid Receptor Profiling Reveals a Low CB1 and High CB2 signature in Clear Cell Renal Cell Carcinoma

**DOI:** 10.1093/oncolo/oyag286.026

**Published:** 2026-08-21

**Authors:** S Singh, S Pattanaik, R S Mavuduru, N Kakkar, G S Bora

**Affiliations:** PhD, Post-Graduate Institute of Medical Education and Research search, Chandigarh - India; Professor, Post-Graduate Institute of Medical Education and Research search, Chandigarh - India; Professor, Post-Graduate Institute of Medical Education and Research search, Chandigarh - India; Professor, Post-Graduate Institute of Medical Education and Research search, Chandigarh - India; Professor, Post-Graduate Institute of Medical Education and Research search, Chandigarh - India

## Abstract

**Background:**

The endocannabinoid system has emerged as a potential regulator of tumour biology; however, the expression and clinical significance of cannabinoid receptors in clear cell renal cell carcinoma (ccRCC) remain poorly understood. We aimed to characterize the expression profile of cannabinoid receptor 1 (CB1) and cannabinoid receptor 2 (CB2) in ccRCC and evaluate their association with clinicopathological features and clinical outcomes.

**Methods:**

A total of 90 patients with histologically confirmed ccRCC were included, comprising a retrospective cohort (n  =  60) and a prospective cohort (n  =  30). Immunohistochemistry was performed to evaluate CB1 and CB2 expression in tumour and adjacent normal renal tissues using staining intensity, percentage positivity, and immune-reactive score (IRS). Protein and transcript-level validation was performed in the prospective cohort using Western blotting and quantitative real-time PCR. Associations with clinicopathological parameters and survival outcomes were analysed using Fisher's exact test and Kaplan-Meier survival analysis.

**Results:**

Tumour tissues demonstrated a distinct cannabinoid receptor signature characterised by reduced CB1 expression and increased CB2 expression compared with adjacent normal kidney tissue (Figure 1). CB1 IRS was significantly lower in tumour samples (P  =  0.001), whereas CB2 IRS was significantly elevated (P < 0.001). Western blot analysis confirmed reduced CB1 protein expression (P  =  0.0466) and increased CB2 protein expression (P  =  0.0283) in tumour tissues. At the transcriptional level, CB2 mRNA expression was significantly upregulated in tumours (P  =  0.0070), while CB1 mRNA expression showed no significant difference (P  =  0.8830), suggesting post-transcriptional regulation of CB1. High CB2 expression was associated with male sex (P  =  0.010) and higher tumour grade (P  =  0.040). No significant associations were observed between CB1 expression and clinicopathological variables. Neither receptor demonstrated independent prognostic significance for overall survival.

**Conclusions:**

ccRCC exhibits a reproducible low CB1/high CB2 molecular phenotype. The selective overexpression of CB2 in tumour tissue highlights its potential as a biomarker and candidate therapeutic target in renal cell carcinoma.

**IUC26690-88 Figure 1:**
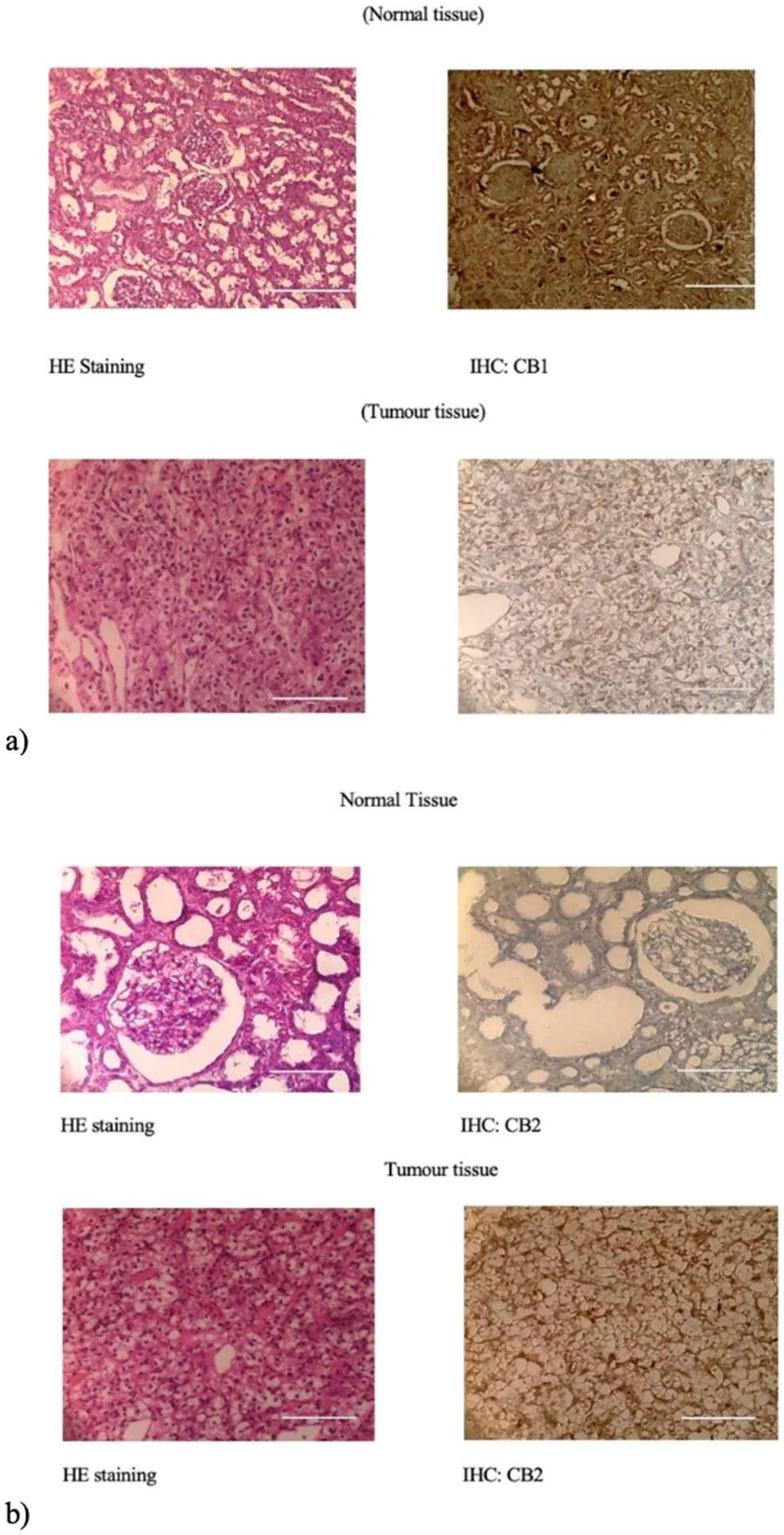
**Differential expression of cannabinoid receptors in clear cell RCC:** a) CB1 expression. b) CB2 expression in normal kidney and tumour tissue.

